# Sodium thiocyanate treatment attenuates atherosclerotic plaque formation and improves endothelial regeneration in mice

**DOI:** 10.1371/journal.pone.0214476

**Published:** 2019-04-02

**Authors:** Andreas Zietzer, Sven Thomas Niepmann, Bakary Camara, Monika Anna Lenart, Felix Jansen, Marc Ulrich Becher, René Andrié, Georg Nickenig, Vedat Tiyerili

**Affiliations:** Medical Department II, University Hospital Bonn, Bonn, Germany; Ludwig-Maximilians-Universitat Munchen, GERMANY

## Abstract

**Introduction:**

Atherosclerotic plaque formation is an inflammatory process that involves the recruitment of neutrophil granulocytes and the generation of reactive oxygen species (ROS). ROS formation by myeloperoxidase, a key enzyme in H_2_O_2_ degradation, can be modulated by addition of sodium thiocyanate (NaSCN). However, the therapeutic use of NaSCN to counteract atherogenesis has been controversial, because MPO oxidizes NaSCN to hypothiocyanous acid, which is a reactive oxygen species itself. Therefore, this study aimed to investigate the effect of NaSCN treatment on atherogenesis *in vivo*.

**Methods:**

Apolipoprotein E knockout (ApoE^−/−^) mice on western-diet were treated with NaSCN for 8 weeks. Blood levels of total cholesterol, IL-10, and IL-6 were measured. Aortic roots from these mice were analyzed histologically to quantify plaque formation, monocyte, and neutrophil granulocyte infiltration. Oxidative damage was evaluated via an L-012 chemiluminescence assay and staining for chlorotyrosine in the aortic walls. Endothelial function was assessed by use of endothelium-dependent vasodilation in isolated aortic rings. Neointima formation was evaluated in wild-type mice following wire injury of the carotid artery.

**Results:**

NaSCN treatment of ApoE^-/-^ mice lead to a reduction of atherosclerotic plaque size in the aortic roots but had no effect on monocyte or granulocyte infiltration. Serum levels of the pro-inflammatory cytokine IL-6 decreased whereas anti-inflammatory IL-10 increased upon NaSCN treatment. In our experiments, we found oxidative damage to be reduced and the endothelial function to be improved in the NaSCN-treated group. Additionally, NaSCN inhibited neointima formation.

**Conclusion:**

NaSCN has beneficial effects on various stages of atherosclerotic plaque development in mice.

## Introduction

Cardiovascular disease (CVD) is the number one cause of global mortality [1]. CVD is caused by atherosclerotic plaques, which are formed through a primarily inflammatory remodeling process of the arterial wall. This process leads to chronic vascular stenoses and ischemic end organ damage [2]. Early stages of arterial remodeling include endothelial dysfunction, invasion of immune cells into the vessel wall, as well as formation of reactive oxygen species (ROS) [3]. In this context, myeloperoxidase (MPO), a heme-enzyme that is produced and secreted by neutrophil granulocytes, promotes ROS formation [4]. MPO is enriched in atherosclerotic plaques and levels of MPO in the plasma can predict cardiovascular mortality following coronary angiography in humans [5][6].

With respect to the molecular mechanism of ROS formation, the heme group of MPO typically serves as an acceptor of electrons during the degradation of H_2_O_2,_ and in turn oxidizes Cl^-^ and Br^-^ ions to HOCl and HOBr via the halogenation cycle [7]. The resulting HOCl is a potent oxidant, which oxidizes high-density (HDL) and low-density lipoproteins (LDL), impairs endothelial function and nitric oxide (NO) production, and induces endothelial apoptosis [8–11].

Recently, our group has shown that pharmacological inhibition of MPO by 4-amino benzoic acid hydrazide (4-ABAH) reduces plaque formation in apolipoprotein E knockout (ApoE^-/-^) mice and decreases neointima formation following carotid injury [12]. Besides 4-ABAH, which inhibits MPO and HOCl production irreversibly, thiocyanate (SCN^-^) has a higher affinity to MPO than Cl^-^ and competitively inhibits HOCl formation through oxidation of SCN^-^ to hypothiocyanous acid (HOSCN) [13,14]. Interestingly, the product of this reaction, HOSCN, has also been reported to oxidize HDL and LDL and to impair endothelial function [15–17]. In contrast to HOCl, HOSCN can be degraded specifically via thioredoxin reductase, which gives HOSCN a more favorable oxidative profile than HOCl [18,19].

In human studies, high blood levels of SCN^-^ after myocardial infarction have been shown to improve long term survival [20]. In contrast, smokers, who are typically at risk for the early development of cardiovascular diseases, also exhibit increased blood levels of SCN^-^ [21]. In order to explore the mechanisms of these conflicting results, we conducted a study, in which we evaluated the effect of SCN^-^ on atherogenesis and its underlying pathological mechanisms in a murine model.

## Methods

### Animals and procedures

**Fig 1:**
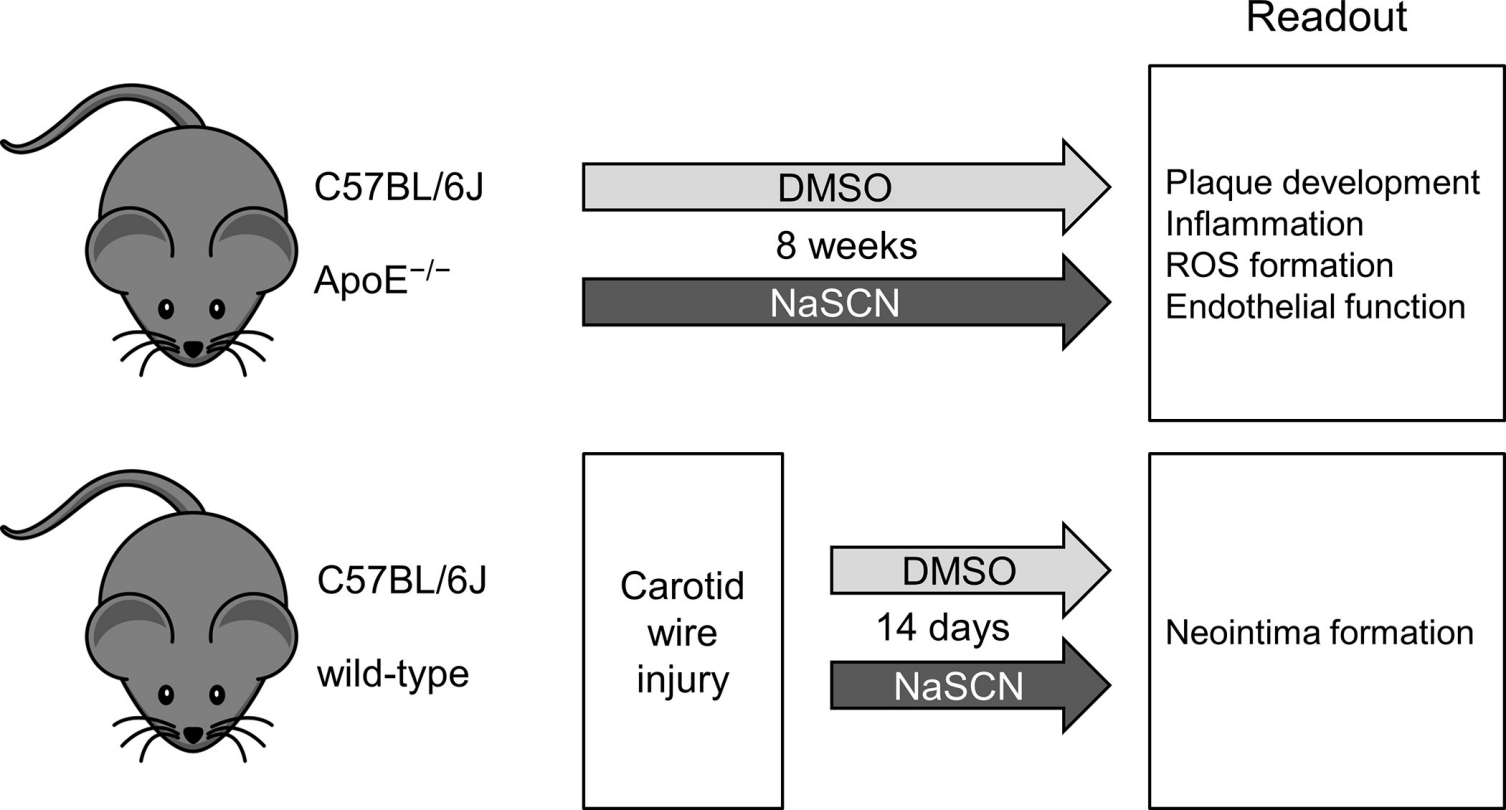
Schematic illustration of the experimental design. For the evaluation of plaque development, inflammation, ROS formation, and endothelial function ApoE^-/-^ mice were used after 8 weeks of intraperitoneal application of NaSCN (200 μg every other day) or vehicle (DMSO). For the assessment of neointima formation wild-type mice were subjected to carotid artery wire injury, treated with NaSCN (200 μg every other day) or vehicle for 14 days, and then analyzed.

For the *in vivo* experiments, we used apolipoprotein E knockout (ApoE^−/−^) and wild-type mice (6–8 weeks old, C57BL/6J background from Charles River, Germany). The animals were kept under standard conditions at 22°C and had free access to water. ApoE^-/-^ mice were fed with a modified western diet consisting of 21% fat, 19.5% casein, and 1.25% cholesterol (SNIFF, Germany). In the intervention group 6 ApoE^-/-^ animals were treated with 200 μg NaSCN in 10% DMSO every second day intraperitoneally, whereas control animals only received DMSO or PBS treatment. This procedure typically leads to a rapid, approximately 2-fold, increase in plasma SCN^-^ levels of the animals, whereas the plasma through levels remain unchanged (S1 Fig). Arterial blood pressure and heart rate were measured with a CODA 6 channel system (Kent Scientific). After 8 weeks of NaSCN / DMSO treatment, the animals were euthanized to collect blood samples and to conduct histological and functional analyses (Fig 1). All animal experiments were carried out in accordance with the animal protection law stated in the German civil code. All investigations were approved by the National Office for Nature, Environment and Consumer Protection in Recklinghausen, Nordrhein-Westfalen (Permit Number: 84–02.04.2013.A390).

### Histological analysis of atherosclerotic lesions

The harvested mouse hearts, including the aortic root, were embedded in Tissue Tek OCT (Miles, U.S.A.) and sliced into 9 μm sections on a Leica cryostat. The aortic sections were placed on poly-L-lysine coated slides and fixed with 3.7% formaldehyde. Subsequently, we stained the sections with oil red O and hematoxylin following standard protocols. Finally, 15 consecutive sections per animal were analyzed by use of a Zeiss Observer microscope (Carl Zeiss, Germany). Digital images were acquired with an AxioCam MRc5 and the Zeiss ZEN imaging software. Atherosclerotic plaque formation was quantified as oil red stained lipid area in percent of the total surface of the vessel wall. Monocyte/macrophage staining was performed by use of an anti-Macrophages/Monocytes antibody, clone MOMA-2 (Merck KGaA, Germany) with an indirect immunoenzymatic staining method. For staining of neutrophil granulocytes, we used a rat anti-neutrophil primary antibody (Abcam, U.K.) and a Cy3-labeled donkey anti-rat-IgG secondary antibody (Jackson Immuno Research Laboratories Inc, U.S.A.). For quantification of HOCl damage, immunohistological staining of chlorotyrosine was performed by use of a rabbit anti-chlorotyrosine antibody (Hycult Biotech, HP5002, Netherlands) and a Cy3-labeled goat anti-rabbit-IgG secondary antibody (Dianova, Germany). As a positive control for the chlorotyrosine staining, aortic sections were pretreated with 0.005% sodium hyopchlorite for 1 hour before staining. A negative control was performed without the primary anti-body.

### Biochemical analysis of plasma cytokine, MPO, and cholesterol levels

Plasma levels of IL-10 and IL-6 were assessed via ELISA by use of a commercially available kit (SA Biosciences, Germany). Measurements were performed in accordance with the manufacturer’s instructions. First, the samples were transferred into antibody-coated wells. After 120 min of incubation at room temperature, unbound sample was washed away and the detection antibodies were added, followed by 60 min of incubation at 37°C. Subsequently, the samples were incubated with Avidin-HRP and the development reagent for 30 min in the dark. Absorbance was measured using a photometer (Tecan Austria, Austria). MPO plasma levels were measured by use of a Mouse MPO ELISA Kit (Abcam, U.K.). The measurements were performed in duplicate, exactly as advised by the manufacturer, by using 75 μL of EDTA Plasma. Blood cholesterol levels were assessed by use of gas chromatography–flame ionization detection.

### Quantification of plasma SCN^-^ levels after injection

Plasma levels of SCN^-^ were quantified in duplicate by use of a colorimetric assay as previously published [22]. In brief, 25 μL EDTA Plasma were diluted 1:3 in water and then 125 μL of 40 mM iron (III) nitrate nonahydrate in 270 mM nitric acid were added. After a 10 min incubation, absorption was measured at 492 nm with a reference of 620 nm in a photometer (Tecan Austria, Austria). Subsequently, 25 μL of 60 mM mercury (II) nitrate monohydrate in 150 mM nitric acid were added, and the absorption measurement was performed again after 5 min. Concentrations were calculated as the difference between the two measurements by use of a standard row of serial dilutions of ammonium thiocyanate.

### Assessment of vascular ROS formation

For evaluation of ROS formation, superoxide release was measured in aortic segments by use of an L-012 chemiluminescence assay. For this purpose, aortic segments were bathed in modified Krebs-HEPES buffer as previously described [12]. Aortas were cut into slices of 2 mm and transferred into scintillation vials, which contained modified Krebs-HEPES buffer as well as 100 mol/L of L-012 (FUJIFILM Wako Chemicals Europe, Germany). After 5 min of incubation, chemiluminescence was measured by use of a scintillation counter (Lumat LB 9501, Berthold) at 1-min intervals over 15 min. Subsequently, the dry weight of the aortic segments was determined in order to calculate the chemiluminescence per mg of tissue.

### Evaluation of endothelial function

In order to assess endothelial function following NaSCN treatment, the descending aortae of ApoE^-/-^ mice were removed and sliced into rings of 3 mm thickness. The aortic rings were immersed into a bath of Tyrode buffer, which was modified as previously described [12]. The bath was warmed to 37°C and continuously aerated with a mixture of 5% CO_2_ and 95% O_2_. In order to record vascular tension, the aortic sections were fixed to a force transducer After extension to a resting tension of 10 mN, increasing concentrations of drugs were added to assess cumulative concentration–response curves: phenylephrine to establish maximal possible contraction (1 nmol/L—100 μmol/L,), carbachol endothelial-dependent (1 nmol/L—100 μmol/L), and nitroglycerin endothelial-independent (1 nmol/L—10 μmol/L).

### Analysis of neointima formation

For the evaluation of neointima formation (n = 10), 6- to 8-week-old wild-type (C57BL/6J, Charles River, Germany) mice were randomized to receive either 200 μg NaSCN in 10% DMSO every second day intraperitoneally or vehicle (10% DMSO) (See Fig 1). For the carotid injury model, the mice were anesthetized with 150 mg/kg body weight ketamine hydrochloride (Ketanest, Pharmacia, Sweden) and 0.1 mg/kg body weight xylazine hydrochloride (Rompun 2%, Bayer, Germany). The carotid injury was performed as previously described by Laufs *et al*. [23]. In brief, the external carotid artery was exposed and ligated distally. Subsequently, the common and internal carotid arteries were occluded in order to allow the insertion of a flexible wire into the external carotid artery. The wire was then advanced into the common carotid artery and rotated inside the vessel to cause endothelial damage. Then the proximal occlusions of the common and internal carotid artery were removed to allow normal blood flow in the injured region. Finally, the skin was closed with single sutures. After 14 days, the mice were euthanized and the carotid arteries were removed, fixed, and embedded by use of Tissue Tek OCT (Miles, U.S.A.). The carotid artery was cut into slices of 7 μm and transferred to poly-L-lysine coated slides (Sigma-Aldrich, U.S.A.) for immunohistochemical analysis. Prior to analysis, the cryosections were postfixed in 4% formaldehyde for 2 min and subsequently incubated in 5% normal goat serum for blocking (Sigma-Aldrich, U.S.A.) for 30 min. In order to assess neointima formation, a fluorescein isothiocyanate-conjugated anti-α-smooth-muscle antibody (Sigma-Aldrich, U.S.A.) was used and 4,6-diamidino-2-phenylindole (DAPI, Sigma-Aldrich, U.S.A.)) was used to stain the nuclei. Additionally, we performed hematoxylin/eosin staining. The sections were analyzed by use of a Zeiss Oberserver microscope (Carl Zeiss, Germany). Digital images were acquired with an AxioCam MRc5 and analyzed with the Zeiss ZEN software.

### Statistical analysis

Statistical analyses were performed using the software GraphPad Prism 7.02 (GraphPad Software, Inc.). Data are presented as the mean ± SEM. Differences between groups were tested for statistical significance by use of an unpaired t-Test or ANOVA followed by Tukey post-hoc analysis (applies for histological analysis of plaque formation and NaSCN plasma levels). A p-value < 0.05 was considered statistically significant.

## Results

### NaSCN treatment leads to decreased atherosclerotic plaque formation in ApoE-/- mice

Treatment of ApoE^-/-^ mice with the MPO-inhibitor NaSCN did not cause a significant difference in serum cholesterol levels (1098 ± 58 mg/dl vs 1051 ± 98 mg/dl, n = 4–6, p = 0.72). We observed a non-significant trend towards a lower heart rate (709.2 ± 20.8 bpm vs. 642.5 ± 38.3 bpm, n = 4–6, p = 0.41) and lower systolic blood pressure (138.2 ± 2.4 mmHg vs 118.1 ± 7.3 mmHg, n = 4–6, p = 0.06) Table 1.

**Table 1:** Blood pressure, body weight, heart rate and cholesterol upon NaSCN treatment.

	Vehicle (DMSO)	NaSCN	p-value
**Body weight [g]**	26 ± 8	25 ± 7	0.928
**Blood pressure [mmHg]**			
**- systolic**	138.24 ± 2.34	118.08 ± 7.26	0.061
**- diastolic**	96.24 ± 3.10	81.96 ± 7.17	0.163
**- mean**	109.90 ± 2.79	93.66 ± 7.11	0.114
**Heart rate [bpm]**	709.16 ± 20.76	642.50 ± 59.47	0.407
**Total serum cholesterol [mg/dl]**	1098 ± 58	1051 ± 98	0.729

**Fig 2:**
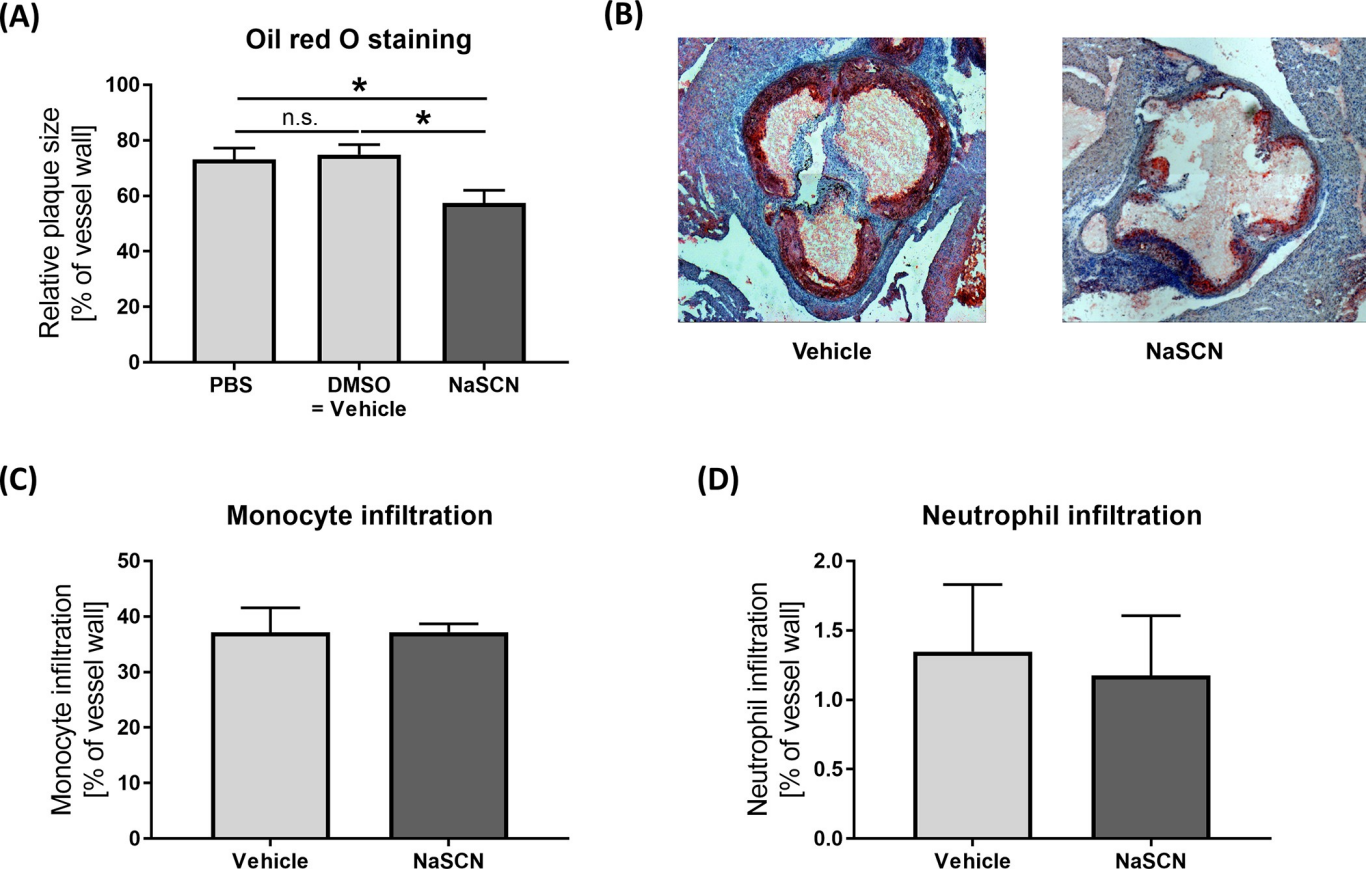
Assessment of atherosclerotic plaque formation, monocyte, and neutrophil granulocyte infiltration in ApoE^-/-^ mice upon NaSCN treatment or DMSO as vehicle. (A) Quantitative analysis of plaque size as a percentage of the total aortic root vessel wall by oil red staining, n = 5–6. (B) Representative histological images of the aortic root (oil red + hematoxylin staining). (C + D) Histological assessment of monocyte and neutrophil granulocyte infiltration as a percentage of the total vessel wall by immunohistological staining, n = 5. Data are presented as the mean ± SEM., *p ≤ 0.05, ***p ≤ 0.005 vs. vehicle.

Histological assessment of atherosclerotic plaque formation by use of oil red staining of the aortic root revealed significantly decreased atherosclerotic plaque size upon treatment with NaSCN compared to DMSO or PBS (PBS 73.09 ± 4.10, DMSO: 74.84 ± 3.62, NaSCN: 57.50 ± 4.51, as a percentage of the total vessel wall, n = 5–6, PBS vs NaSCN p = 0.047, DMSO vs NaSCN p = 0.027) (Fig 2). As MPO is typically released by macrophages and neutrophil granulocytes, we measured the infiltration of those two cell types by immunohistological staining. No significant difference in monocyte or neutrophil granulocyte infiltration was detected between the NaSCN-treatment group and the control group, n = 5, (Fig 2).

### NaSCN improves inflammatory cytokine levels in the plasma as well as ROS and chlorotyrosine formation in the vessel wall

**Fig 3:**
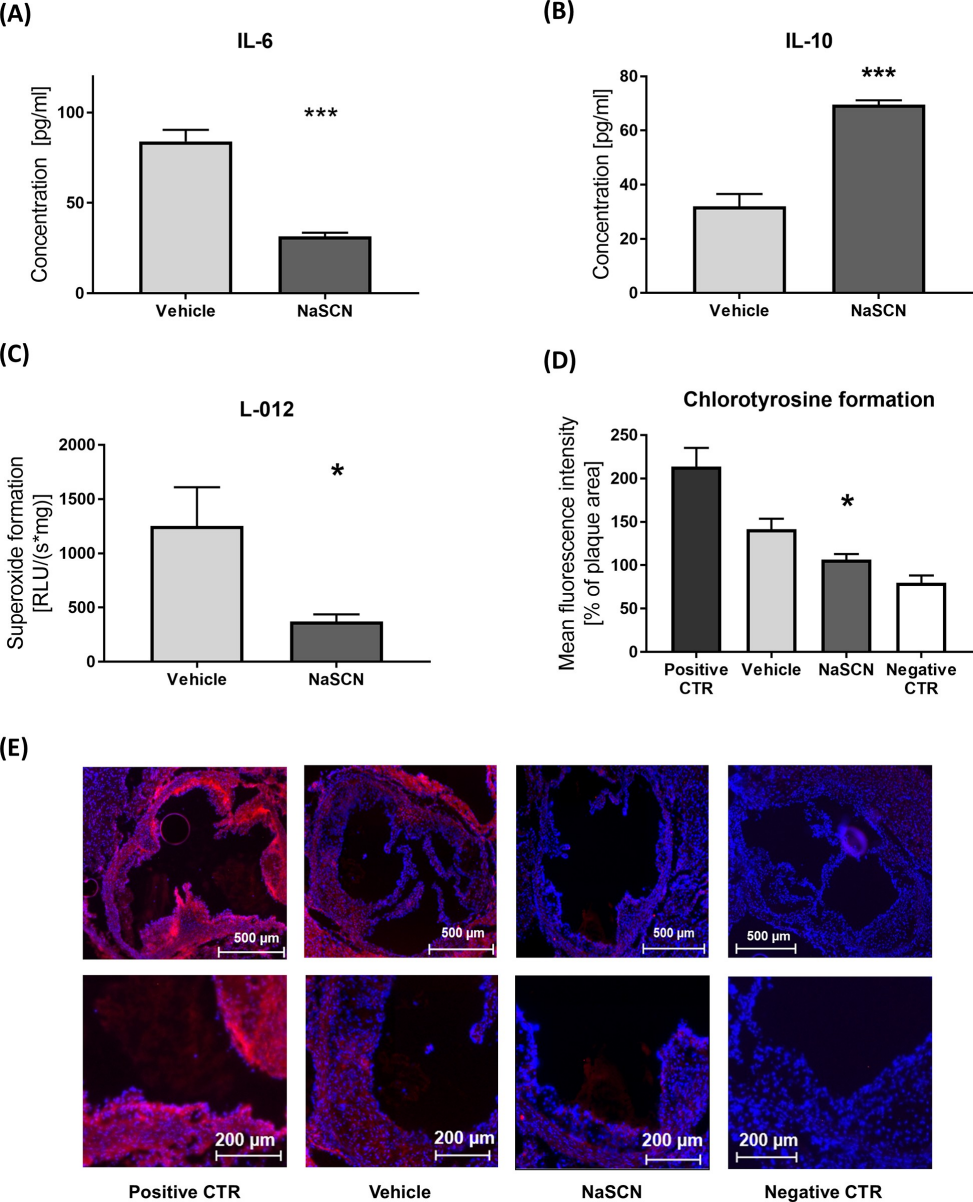
Assessment of IL-6 and IL-10 plasma levels and ROS / chlorotyrosine formation in ApoE^-/-^ mice upon NaSCN treatment or DMSO as vehicle. (A + B) Plasma IL-6 and IL-10 levels upon NaSCN treatment measured by ELISA, n = 4. (C) Measurement of ROS formation in aortic segments by L-012 chemiluminescence, n = 4. (D) Quantification of HOCl-dependent tissue damage via immunohistological staining of 3-cholortyrosine in the atherosclerotic plaque area, n = 4. (E) Representative images of Chlorotyrosine staining, positive control after incubation with 0.005% HOCl for 1 hour, negative control only with secondary anti-body (Red chlorotyrosine, Blue DAPI). Data are presented as the mean ± SEM., n = 4, *p ≤ 0.05, ***p ≤ 0.005 vs. vehicle.

In order to quantify the effect of NaSCN treatment on vascular inflammatory processes, which underlie atherosclerotic plaque formation, plasma cytokine levels were quantified by ELISA. The plasma levels of the pro-inflammatory IL-6 were significantly reduced (84.04 ± 6.37 pg/ml vs 31.52 ± 2.06 pg/ml, n = 4, p = 0.0002) in NaSCN-treated mice, whereas levels of the anti-inflammatory IL-10 were increased in the NaSCN-treatment group (31.98 ± 4.56 pg/ml vs 69.50 ± 1.69 pg/ml, n = 4, p = 0.0002) (Fig 3). In order to evaluate if NaSCN treatment has an effect on MPO secretion into the blood, we measured MPO plasma levels by ELISA. No difference in MPO plasma levels was detected between the NaSCN treatment group and the vehicle group (S2 Fig).

Subsequently, we analyzed ROS formation in the aortic segments. We hypothesized that by decreasing the amount of HOCl produced by MPO upon NaSCN treatment, local oxidative stress levels would be reduced. Quantification of L-012 chemiluminescence in the aortic segments showed a significant reduction of ROS formation upon treatment with NaSCN 1252.9 ± 356.9 vs 370.1 ± 67.1 RLU/(s*mg), n = 4–6, p = 0.017 (Fig 3). In order to assess specific HOCl dependent oxidative damage in the aortic tissue, we assessed chlorotyrosine formation immunohistologically in the atherosclerotic plaque area. Chlorotyrosine formation was significantly reduced in the NaSCN treatment group (Fig 3).

### Endothelium-dependent vasodilation is augmented upon NaSCN treatment

**Fig 4:**
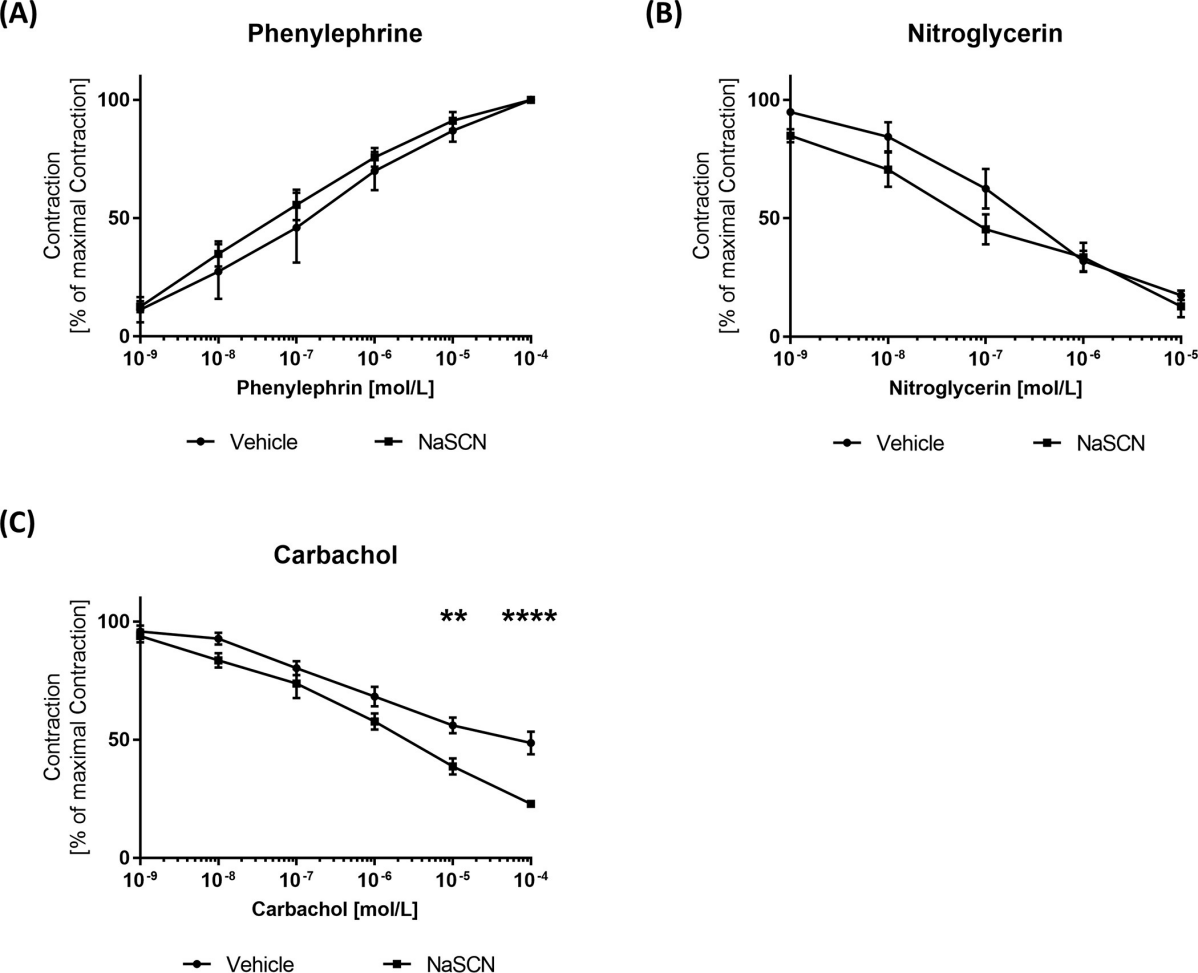
Measurement of endothelial function in isolated aortic segments of ApoE−/− mice upon NaSCN treatment in organ chamber experiments. (A) Definition of the maximal endothelial contraction by incubation with increasing phenylephrine concentrations. (B) Assessment of endothelium-independent vasodilation as a percentage of the maximal contraction with increasing concentrations of nitroglycerin. (C) Assessment of endothelium-dependent vasodilation as a percentage of the maximal contraction with increasing concentrations of carbachol. Data are presented as the mean ± SEM, n = 4–5, **p ≤ 0.01, ***p ≤ 0.005 vs. vehicle.

As a next step, we assessed the effect of NaSCN on endothelial function. Therefore, aortic rings were isolated from ApoE^-/-^ mice after 8 weeks of treatment with NaSCN and transferred to organ baths to measure cumulative concentration–response curves. First, maximal contraction was established upon phenylephrine treatment (Fig 4). Then in order to test vasodilation, isometric tension was measured upon incubation with carbachol and nitroglycerine. Endothelium-dependent vasodilation induced by 100 μmol/L of carbachol was significantly enhanced by NaSCN treatment (Vehicle 56.02 ± 3.37% vs NaSCN 22.88 ± 1.56% of maximum dilation, n = 4–5, p<0.0001). In contrast, testing for endothelium-independent vasodilation by use of 10 μmol/L nitroglycerin showed no significant effect of prior MPO inhibition by NaSCN (17.44 ± 2.06% vs 12.76 ± 4.60% of maximum dilation, n = 4, p = 0.39) (Fig 4).

### Neointima formation is alleviated after NaSCN treatment

**Fig 5:**
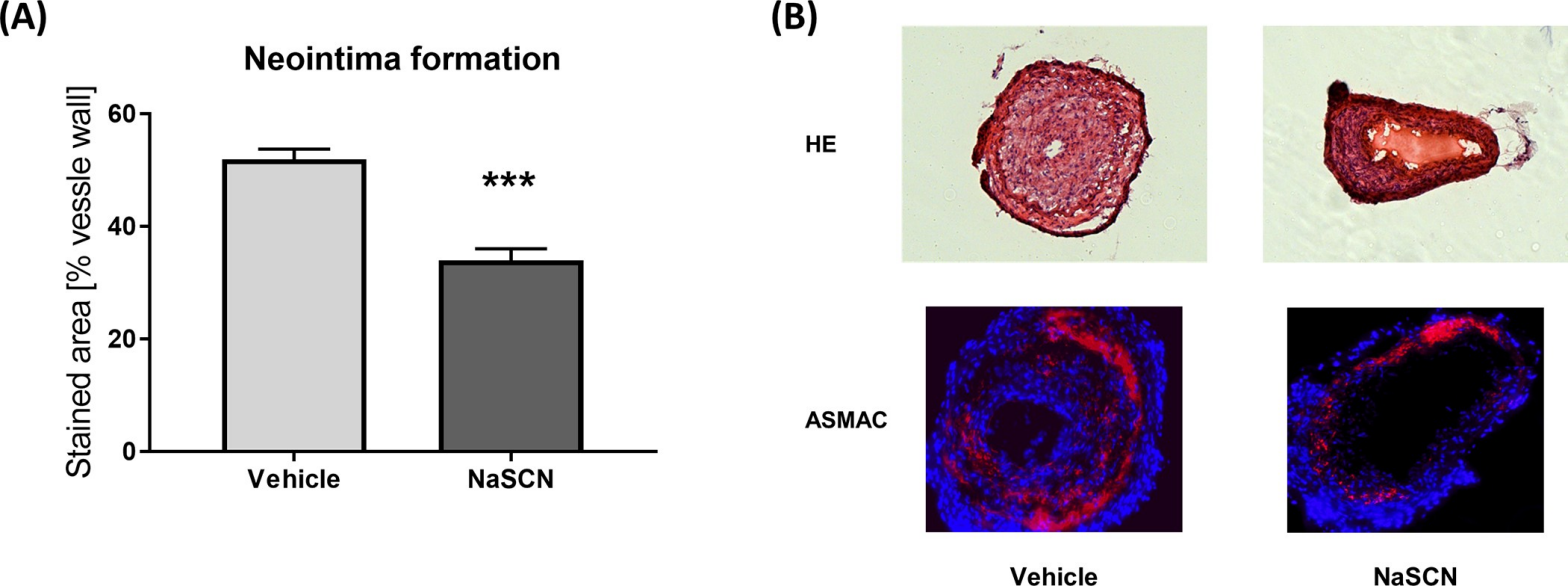
Evaluation of neointima formation after carotid-artery wire injury in wild-type mice upon NaSCN treatment. (A) Quantitative analysis of neointima formation by use of hematoxylin/eosin staining as a percentage of the vessel wall. (B) Representative histological images of the injured carotid artery 14 days post injury. Upper panel with hematoxylin/eosin staining. Lower panel with anti-α-smooth-muscle Actin staining (red) and DAPI (blue). Data are presented as the mean ± SEM, n = 5, ***p ≤ 0.005 vs. vehicle.

After having evaluated the effects of NaSCN on the early stages of atherosclerotic plaque formation, we focused on neointima formation, which typically occurs after prolonged endothelial damage, in the later stages of CVD. Therefore, we conducted carotid artery wire-injury experiments in wild-type mice and quantified neointima formation histologically by hematoxylin/eosin staining and anti-α-smooth-muscle actin staining 14 days post injury. The neointima was quantified from the hematoxylin/eosin stained sections. We detected a significant reduction of neointima formation after carotid wire injury upon treatment with NaSCN: 51.98 ± 1.78 vs. 34.03 ± 2.06 neointima area as a percentage of the total vessel wall according to hematoxylin/eosin staining, n = 5, p = 0.0002 (Fig 5).

## Discussion

In this study, we provide evidence that NaSCN treatment reduces atherosclerotic plaque formation in a mouse model of CVD. This effect appears to be independent of monocyte infiltration and blood LDL levels. NaSCN had positive effects on the inflammatory status of the animals, as plasma levels of the pro-inflammatory cytokine IL-6 were reduced upon NaSCN treatment whereas the anti-inflammatory cytokine IL-10 levels were increased. Locally, oxidative stress, an early surrogate marker of endothelial damage, was reduced in the vascular tissue. In line with these results, endothelial function was better preserved in the NaSCN-treated group. Additionally, we show that NaSCN treatment reduces neointima formation following endothelial damage.

The role of thiocyanate treatment to prevent atherosclerosis has been a controversial topic. Our findings are in line with a previous study from Morgan *et al*., who suggest a beneficial effect of oral thiocyanate supplement on atherosclerotic plaque formation in LDL-receptor knock-out mice [24]. NaSCN has a high affinity for MPO and is therefore able to competitively inhibit the formation of HOCl and HOBr at the expense of increased HOSCN formation [14]. HOSCN is also a potent oxidant and mediates various negative cellular effects, such as the disturbance of macrophage glucose metabolism [25] and of nitric oxide production in endothelial cells [15]. But in contrast to HOCl and HOBr, HOSCN can be effectively detoxified by human thioredoxin reductase [19]. Consequently, NaSCN reduces the oxidative capacity of MPO, leading to reduced oxLDL formation *in vitro* [26].

In previous studies, a direct inhibition of MPO via 4-aminobenzoic acid hydrazide (4-ABAH) or other small molecules has been found to decrease the development of atherosclerosis [12,27]. Compared to previous results from our own group the effects of 4-ABAH and NaSCN are similar in terms of plaque reduction, inflammatory response, endothelial function, and neointima formation [12].

In the present study, we have applied two different methods to quantify oxidative stress and oxidative damage locally in the vessel wall. The L-012 chemiluminescence assay has originally been developed to specifically measure NADPH oxidase-derived superoxide. The transformation of L-012, however, not only depends on superoxide formation, but is also sensitive to peroxidase activity [28]. Therefore, we used the L-012 as an unspecific indicator of oxidative stress in the tissue, as suggested by Daiber *et al*. [29]. In order to specifically quantify HOCl mediated oxidative damage, we applied an immunohistological staining method to assess the formation of chlorotyrosine residues in the atherosclerotic plaque area.

Interestingly, we found a trend towards decreased blood pressure in our NaSCN group compared to vehicle treatment. This can be explained by the vasoactive cyanate component, which has be used as an anti-hypertensive drug in the form of potassium thiocyanate and is still used as an emergency anti-hypertensive drug in the form of sodium nitroprusside [30].

In summary, our study provides a comprehensive analysis of the effect of thiocyanate on the relevant stages of atherosclerosis development. NaSCN treatment was found to affect all stages of plaque formation positively, including inflammatory processes, oxidative damage, endothelial function, and neointima formation. In conclusion, the general profile of NaSCN treatment to reduce atherogenesis seems to be positive in mice.

## Supporting information

S1 Dataset Underlying data to all figures. (XLSX)

S1 Fig NaSCN plasma levels. A) Time course of NaSCN plasma levels before, 15 min, 1 h, and 6 h after injection, n = 3/4, *p ≤ 0.05, **p ≤ 0.05 B) NaSCN plasma levels of vehicle and NaSCN treated animals at sacrifice, 48 h after the last injection. Data are presented as the mean ± SEM, n = 5.
(TIF)

S2 Fig MPO plasma levels. MPO Plasma levels in the vehicle and NaSCN treatment group, Data are presented as the mean ± SEM, n = 4.
(TIF)
