# Supplementary figures and images for: Sodium thiocyanate treatment attenuates atherosclerotic plaque formation and improves endothelial regeneration in mice
Source: PLoS One. 2019 Apr 2;14(4):e0214476. doi: 10.1371/journal.pone.0214476 (PMC6445437; doi:10.1371/journal.pone.0214476)

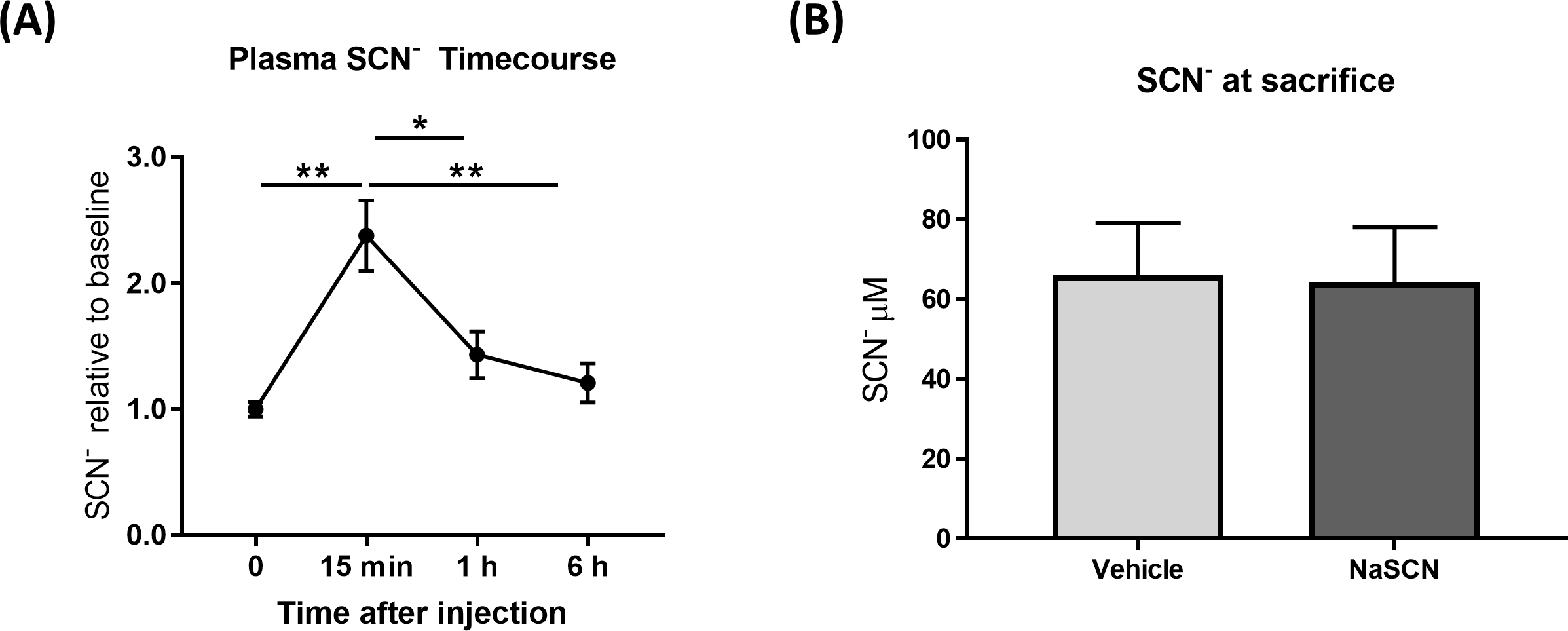

Supplement: S1 Fig — A) Time course of NaSCN plasma levels before, 15 min, 1 h, and 6 h after injection, n = 3/4, *p ≤ 0.05, **p ≤ 0.05 B) NaSCN plasma levels of vehicle and NaSCN treated animals at sacrifice, 48 h after the last injection. Data are presented as the mean ± SEM, n = 5. (TIF) [file pone.0214476.s002.TIF]

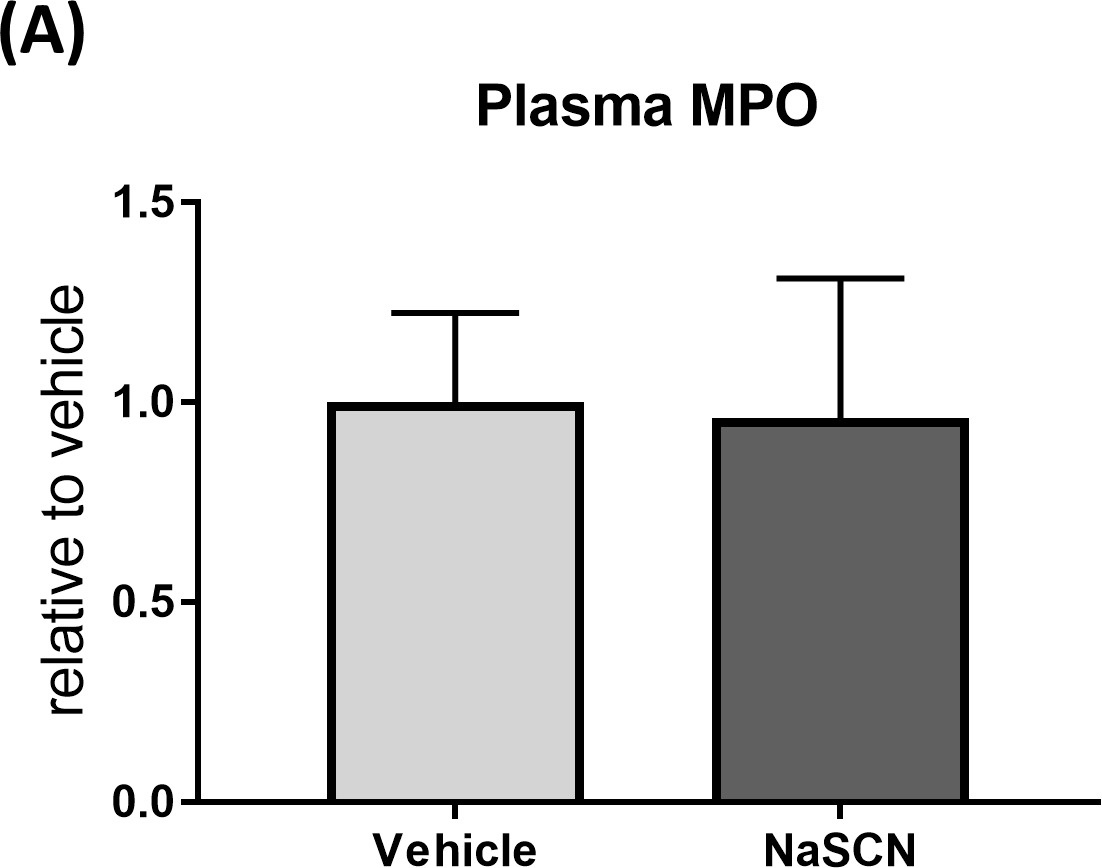

Supplement: S2 Fig — MPO Plasma levels in the vehicle and NaSCN treatment group, Data are presented as the mean ± SEM, n = 4. (TIF) [file pone.0214476.s003.tif]
